# Adaptation by death? Cell death-based tolerance to cadmium in 150-generation exposure of *Spodoptera exiqua* Hübner (Lepidoptera: Noctuidae)

**DOI:** 10.1093/ee/nvad077

**Published:** 2023-10-06

**Authors:** Agnieszka Babczyńska, Magdalena Rost-Roszkowska, Alina Kafel, Bartosz Łozowski, Maria Augustyniak, Monika Tarnawska

**Affiliations:** Institute of Biology, Biotechnology and Environmental Protection, University of Silesia in Katowice, Bankowa 9, 40-007 Katowice, Poland; Institute of Biology, Biotechnology and Environmental Protection, University of Silesia in Katowice, Bankowa 9, 40-007 Katowice, Poland; Institute of Biology, Biotechnology and Environmental Protection, University of Silesia in Katowice, Bankowa 9, 40-007 Katowice, Poland; Institute of Biology, Biotechnology and Environmental Protection, University of Silesia in Katowice, Bankowa 9, 40-007 Katowice, Poland; Institute of Biology, Biotechnology and Environmental Protection, University of Silesia in Katowice, Bankowa 9, 40-007 Katowice, Poland; Institute of Biology, Biotechnology and Environmental Protection, University of Silesia in Katowice, Bankowa 9, 40-007 Katowice, Poland

**Keywords:** autophagy, apoptosis, tolerance

## Abstract

Mechanisms, including autophagy and apoptosis, which serve to regulate and ensure proper organism functions under optimal conditions, play additional defensive roles under environmental pressure. The aim of this study was to test the following hypotheses: (i) elevated autophagy and apoptosis intensity levels, as defensive processes in response to contact with cadmium, are maintained for a limited number of generations and (ii) the number of generations after which levels of cell death processes reach the reference level depends on selective pressure. Cell death processes were assessed by light and transmission electron microscopy, terminal deoxynucleotidyl transferase dUTP nick end labeling(TUNEL), and cytometric analyses. Model insects (*Spodoptera exiqua*, Hübner, 1808) were orally exposed to various concentrations of cadmium for 18 generations and compared with reference strains exposed to cadmium or not (control) for over 150 generations. Elevated programmed cell death intensity levels decreased after several generations, indicating tolerance of individuals to cadmium in the diet and verifying the first hypothesis; however, testing the second hypothesis indicated that the number of generations depended not only on pressure intensity, but also on cell death type, since levels of autophagy remained increased for a minimum of 12 generations.

## Graphical Abstract

**Graphical Abstract:**
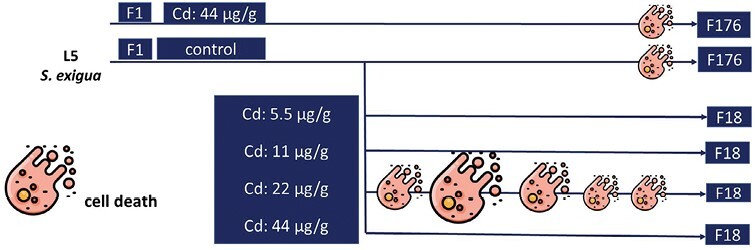

## Introduction

Numerous studies conducted worldwide have demonstrated that environmental pressure induces changes that enable individuals to survive in polluted environments; this is established and has been confirmed for all living organisms. Individuals able to survive and reproduce create populations resistant to specific conditions, in most cases caused by intentional (urbanization, agriculture, etc.) or unintentional (ecological catastrophes) human activity. After several generations of exposure to pollutants, the resulting populations differ from original or reference populations in relation to life history parameters, such as body size or proportions, and duration of ontogenesis or life span, as well as reproduction strategies (Augustyniak et al. 2008, Wilczek et al. 2008, 2013, Babczyńska et al. 2012). These differences suggest that such adaptation is energetically costly and requires reallocation of energy from growth and reproduction to protection and detoxification. The protective processes involved are relatively well recognized. Diverse pathways that eliminate the harmful effects of toxic agents have been described, due to the wide range of mechanisms of toxicity; these include elimination (in feces; Hopkin 1989), neutralization (granules; van der Zande et al. 2020), transformation (enzymes; Pedersen et al. 2020), free radical scavenging (antioxidants; Villada-Bedoya et al. 2021), or cell death (autophagy, apoptosis, and necrosis; Rost-Roszkowska et al. 2020a, 2020b) pathways, that have been the subject of intense recent study. However, many unsolved questions about the basis of such adaptation, namely whether it is genetically preserved and heritable in subsequent generations of the population or results from broad phenotypic plasticity, remain unanswered. This question seems particularly significant in light of, for example, the resistance of pests to plant protection treatments. Solving such questions in situ within ecosystems is highly challenging, due to the complexity of factors acting simultaneously in uncontrolled conditions. Unlike field conditions, laboratory animal populations allow the study of selected factors under controlled conditions in precisely designed experimental groups, comprising individuals with uniform characteristics (e.g., age and sex). A laboratory population fulfilling these requirements is available at the Institute of Biology, Biotechnology, and Environmental Protection, the University of Silesia in Katowice, Poland. It was established in 2006, since when the 1st generation of individuals of the moth, *Spodoptera exigua*, was transferred to a diet supplemented with 44 µg Cd per g dry food weight (Cd strain). Until the year of the experiment presented here, these insects have reproduced for over 150 generations. Studies conducted during that time have suggested that individuals have adapted to the pressure of Cd exposure to create a stable population. The survival and reproduction success of the resulting insects do not differ from those of the original reference (K) strain. Apart from life-history parameters, other biomarkers measured in the Cd strain do not differ from those of the reference strain, indicating that the insects have undergone adaptation (Kafel et al. 2012a, 2012b, Augustyniak et al. 2016, Płachetka-Bożek and Augustyniak 2017, Płachetka-Bożek et al. 2018a, b, Tarnawska et al. 2019, Augustyniak et al. 2020, Babczyńska et al. 2020). This rich data source was the inspiration to design an experiment with the potential to provide data that can conclusively demonstrate whether or not the adaptation of the insects results from phenotypic or genotypic changes. In our experiments, we established new groups of insects receiving the same Cd concentration in their food as the Cd strain, as well as groups receiving intermediate concentrations of Cd. Details of the project have been presented previously by Tarnawska et al. (2019) and Augustyniak et al. (2020). To evaluate hypothetical adaptation stages, we selected universal parameters indicative of protective processes, including levels of stress proteins (Tarnawska et al. 2019) and DNA damage (Augustyniak et al. 2020). The contribution of these parameters to adaptation/tolerance will be discussed elsewhere in the text. We also refer to some aspects of autophagy that were reported in a recent manuscript, addressing the questions of multigenerational and acute exposure to high Cd concentration (Babczyńska et al. 2020). Briefly, after a number of generations, levels of both stress proteins and DNA damage in Cd-exposed insects gradually became similar to those in control individuals. Moreover, the number of generations required to reach parameter levels equivalent to those in the control group was generally negatively correlated with the concentration of cadmium in the insect food (Tarnawska et al. 2019, Augustyniak et al. 2020).

As a developmental process, autophagy is not a major protective pathway; however, it may play an adaptive role under energy deficiency conditions, such as starvation (Lipovšek et al. 2018, Włodarczyk et al. 2019). As it is, autophagy is not beneficial as a protective mechanism; however, it may be induced in response to sudden intense exposure to a stressor (Babczyńska et al. 2020). Depending on the type, strength, and duration of exposure to a stressor, autophagy may be a survival pathway or a type of cell death. In addition, selective or nonselective autophagy can be activated in order to neutralize e.g., damaged organelles, xenobiotics, storage materials, etc. (Chen et al. 2017, Rost-Roszkowska et al. 2019, Tarique et al. 2019). Moreover, clear cross-talk has been described between autophagy and apoptosis, as well as between autophagy and necrosis (Das et al. 2012, Jung et al. 2020). We considered the possibility that autophagy may be initiated only if other defensive mechanisms (e.g., antioxidative functions) are inefficient (Babczyńska et al. 2020).

Unlike autophagy, apoptosis has been investigated in the context of exposure to pollution both in the natural environment and under laboratory conditions. Gautam et al. (2020) presented evidence for increased apoptosis in animals exposed to pollutants in their natural habitat. The authors found an increased level of apoptosis indices in coelomocytes of earthworms from polluted soils if the stress was low or moderate, while under heavy pressure, necrotic cells were dominant. Gautam et al. (2020) concluded that necrosis and apoptosis can be considered as stress biomarkers under the specific conditions investigated (tannery fields). Similar findings were generated from a study of clams from a metal polluted lagoon, and the authors suggested that apoptosis can serve as a biomarker of exposure to anthropogenic changes (Mansour et al. 2020). Laboratory evidence for chemical-induced apoptosis has also been published; for example, Wilczek (2005) reported that the percentage of apoptotic cells in the midgut glands of the spider, *Agelena labyrinthica (*Clerck*,* 1757; Araneae: Agelenidae), was higher in individuals exposed to the pesticide, dimethoate, than that in control spiders. Increased apoptosis parameter levels were also found in response to laboratory copper exposure in the shrimp, *Litopenaeus vannamei* (Guo et al. 2017).

In this study, we aimed to explore the role of cell death processes in the Cd tolerance that has developed in a population of *S. exigua* insects after chronic (over 150 generations) exposure to this metal. The following hypotheses were formulated, based on the results of previous experiments:

Hypothesis 1. Elevated autophagy and apoptosis intensity levels are maintained for a limited number of generations. The intensity of cell death processes reflects the tolerance of insects to cadmium, with lower intensity associated with higher tolerance.

Hypothesis 2. The number of generations after which the level of cell death processes reaches the reference level, after increasing in response to contact with cadmium, depends on selective pressure on individuals, where pressure is stronger when the concentration of cadmium in insect food is higher, and stronger pressure causes a population to become tolerant after fewer generations of exposure.

## Materials and Methods

### Test Animals and Experimental Set-up

Insects used for the experiment came from a laboratory *S. exigua* colony established in 1999 at the Institute of Biology, Biotechnology, and Environmental Protection, University of Silesia in Katowice. In 2005, selection of insects for cadmium resistance was initiated by providing food contaminated with a low concentration of Cd (44 μg Cd per g dry weight), chosen based on a pilot study in which animals were exposed in a range of concentrations between 0 and 704 μg Cd per g of dry weight in food; 44 μg Cd per g of food dry weight was selected as the lowest concentration at which a significant effect on insect development was observed (Babczyńska et al. 2020). A precise description of breeding procedures was reported by (Kafel et al. 2012a, 2012b). The cadmium strain (Cd strain) had been established for 147 generations at the beginning of the present experiment and 164 generations at the end. Six experimental groups were established for the present study, as follows:

Control – strain without cadmium administered in food.

Cd – strain exposed to cadmium (44 μg·g^−1^ of dry food) for 147 generations. Administration of Cd continued unchanged until the 164th generation.

Plus, 4 groups were generated from the control strain by switching to food contaminated with cadmium at the beginning of the experiment. Cd administration was continued for the following 18 consecutive generations. The groups comprised insects given food containing cadmium at 5.5 μg·g^−1^ (Cd5.5), 11 μg·g^−1^ (Cd11), 22 μg·g^−1^ (Cd22), and 44 μg·g^−1^ (Cd44) of dry food.

Measurements were performed in generations 1–6, 12, and 18 in all experimental groups switched to contaminated from control diets, and in equivalent generations 147–152, 158, and 164 in the Cd and control groups.

### Cytometric Analyses

Cells status was analyzed as described previously (Flasz et al. 2021a, 2021b). Briefly, 5 individuals from each experimental group were anesthetized on ice. One of the proleg was cut off and the leaking hemolymph was collected directly to the Eppendorf tube. Then, 40 µl of hemolymph was suspended in phosphate buffer (PBS, 400 μl, pH 7.4, 0.1 M) and homogenized (Minilys, Bertin Technologies). The resulting cell suspensions were analyzed using a Guava Muse Cell Analyzer (Luminex Corp.) with a Muse Annexin V & Dead Cell Kit, to determine percentages of live, dead, and apoptotic cells.

### Light Microscopy and Transmission Electron Microscopy (TEM)

TEM makes it possible to distinguish autophagic structures having, for example, a double membrane formed from a phagophore, as well as to precisely describe the cellular organelles, structures, xenobiotics, and storage materials that are eventually degraded inside the autophagosomes (Yla-Anttila et al. 2009, Bustos et al.2017, Jung et al. 2019, Neikirk et al. 2021). Conventional TEM enables to distinguish of different forms of autophagy: selective and nonselective processes (Chen et al. 2017, Rost-Roszkowska et al. 2019, Tarique et al. 2019).

Autophagic and apoptotic changes were analyzed using light and transmission electron microscopes, and quantitative qualitative analyses of autophagic structures, including autophagosomes, autolysosomes, residual bodies, and lysosomes, were conducted. Fifth larval stage *S. exiqua* specimens were anesthetized with chloroform and their midguts were isolated; 3 specimens from each studied generations of each experimental group were analyzed. Material was fixed with 2.5% glutaraldehyde in 0.1 M sodium phosphate buffer (pH 7.4, 4 °C, 2 h), postfixed in 2% osmium tetroxide in 0.1 M phosphate buffer (4 °C, 2 h), and dehydrated in a graded series of ethanol concentrations (50, 70, 90, 95, and 4 × 100%, each for 15 min) and acetone (15 min). Then, midgut specimens were embedded in epoxy resin (Epoxy Embedding Medium Kit; Sigma-Aldrich, St. Louis, MO, USA). Semi-(0.8 µm thick) and ultra-thin (70 nm) sections were cut on a Leica Ultracut UCT25 ultramicrotome. Semi-thin sections (*n* = 100) were cut from each individual, stained with 1% methylene blue in 0.5% borax, and observed using an Olympus BX60 light microscope. After staining with uranyl acetate (5 min) and lead citrate (5 min), ultra-thin sections were examined using a Hitachi H500 transmission electron microscope at 75kV.

### Quantitative Analysis

Numbers of midgut epithelial cells with autophagic structures (autophagosomes, autolysosomes, and residual bodies) relative to the total number of cells was determined using ultrathin sections; TEM enabled discrimination of columnar (digestive), endocrine, goblet, and regenerative cells (Shokri et al. 2012). Percentages of cells with autophagic structures were determined by counting 100 random cells (columnar, endocrine, goblet, and regenerative cells separately) in midguts from 3 specimens from each experimental group (control, Cd5.5, Cd11, Cd22, Cd44, Cd) at each generation mentioned above. Within these 100 cells, the numbers possessing autophagic structures were counted. Sections were randomly selected from the entire length of the midgut, so that cells from different regions of the organ were always analyzed.

### Cryosections and TUNEL Assay

The TUNEL assay is a sensitive method to detect the DNA fragmentation that occurs during apoptosis; it uses terminal deoxynucleotidyl transferase (TdT) to enable incorporation of deoxynucleotides at the free 3’-hydroxyl ends of fragmented DNA. Isolated midguts from 2 specimens from all examined generations of each experimental group were fragmented and embedded in tissue-freezing medium (Jung) at −22 °C, without fixation. Cryostat sections (5 μm thick) were cut and mounted on 1% gelatin-coated slides (SuperFrost Plus slides, Menzel-Gläser), incubated in permeabilization solution (0.1% sodium citrate, 2 min on ice at 4 °C), washed in Tris-buffered saline (3 × 5 min, room temperature), and stained with TUNEL reaction mixture (In Situ Cell Death Detection Kit, TMR red, Roche), according to the manufacturer’s protocol (60 min in the dark, 37 °C). Slides were analyzed using an Olympus BX60 fluorescence microscope. TdT negative controls were prepared according to the labeling protocol (In Situ Cell Death Detection Kit, TMR red, Roche).

### Statistical Analysis

Statistical analyses were conducted using TIBCO Software Inc. (2017) STATISTICA (data analysis software system). Comparisons were conducted by nonparametric ANOVA and Kruskal–Wallis H tests, applied as posthoc analysis; *P* < 0.05 was considered significant.

## Results

### Ultrastructure of the Midgut Epithelium in *S. exiqua* Larvae

**Fig. 1.**
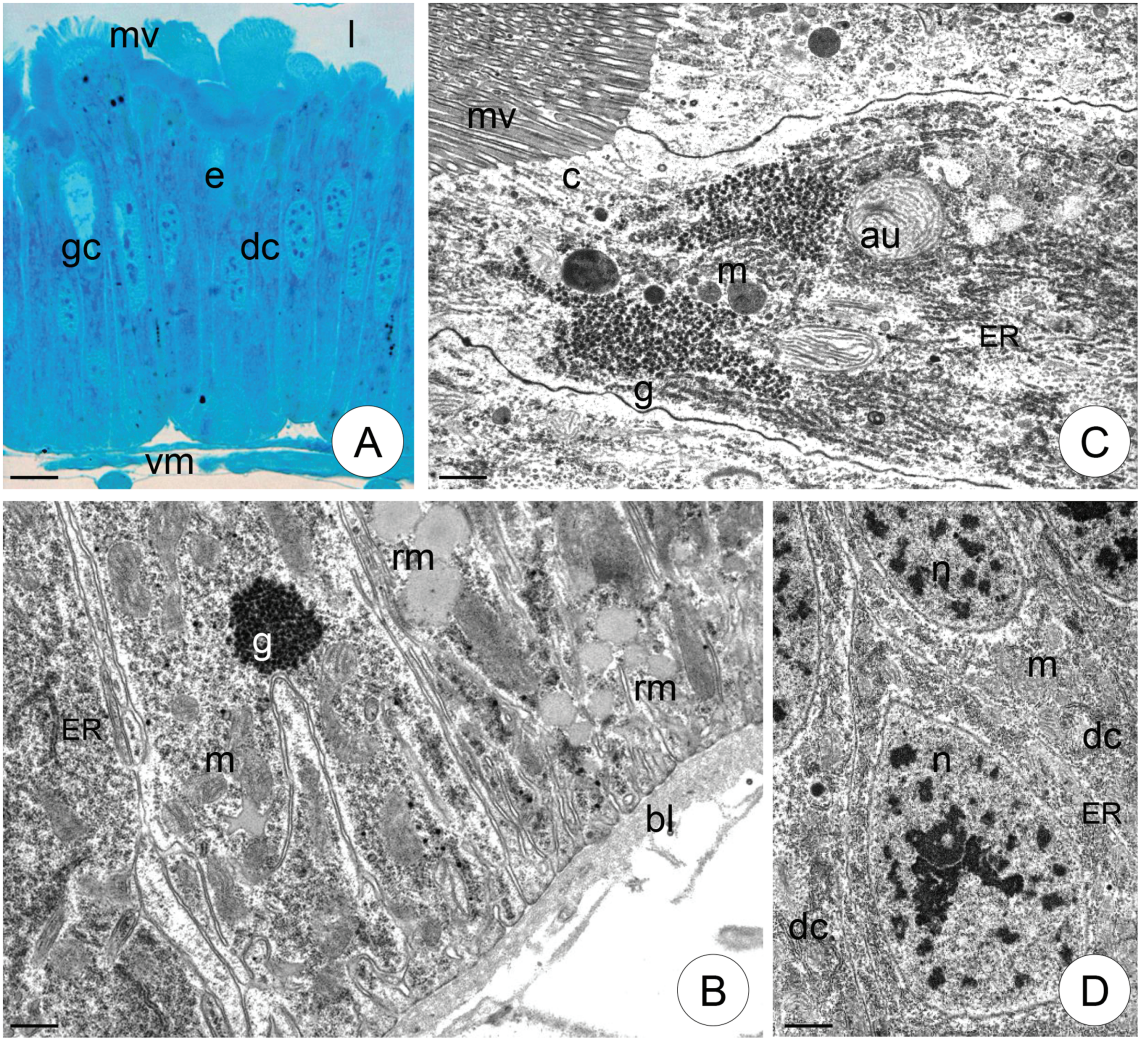
The midgut of V instar larvae of *S. exiqua* in control specimens. (A) Light microscope. Scale bar = 20 µm. (B) The basal cytoplasm of columnar cells. Scale bar = 5 µm. TEM. (C) The apical cytoplasm of columnar cells. Scale bar = 5 µm. TEM. (D) Regenerative cell among basal regions of columnar cells. Scale bar = 5 µm. TEM. Midgut epithelium (e), columnar cells (dc), goblet cells (gc), midgut lumen (l), microvilli (mv), mitochondria (m), nucleus (n), cortical layer (c), autophagosomes (au), endoplasmic reticulum (ER), glycogen granules (g), reserve material (rm), basal lamina (bl), visceral muscles (vm).

In the fifth larval stage of *S. exiqua*, the midgut epithelium is formed by a single layer of epithelial cells, among which columnar, endocrine, and goblet cells are distinguishable (Fig. 1). Single regenerative cells are randomly scattered between basal regions of epithelial cells (Fig. 1D), and there are no differences in epithelium structure along the entire length of the midgut.

The precise structure and ultrastructure of *S. exiqua* larvae have been described in previous papers (e.g., Rost-Roszkowska et al. 2008); thus, only a general description is presented here. The cytoplasm of columnar cells showed distinct regionalization, allowing the basal, perinuclear, and apical regions to be distinguished. The basal cell membrane of columnar cells formed small folds, accompanied by some mitochondria and rough endoplasmic reticulum cisternae (Fig. 1B). The oval nuclei were surrounded by numerous rough and smooth endoplasmic reticulum cisternae, as well as Golgi complexes. The apical cytoplasm had a typical cortex layer, with distinct microvillar cores. Mitochondria, rough endoplasm reticulum cisternae, and glycogen granules were accumulated just beneath the cortical layer (Fig. 1C). Some lipid droplets and spheres with the reserve material are gathered in the cytoplasm (Fig. 1B). Goblet cells (Fig. 1A) contained cavities, formed by the bulging of their apical membranes, and containing microvilli into the extracellular space. Microvilli in such cavities often contained mitochondria; however, microvilli at the apical part of the cavity, reaching the midgut lumen, lacked mitochondria and formed a type of valve that allowed contact of the cell cavity with the midgut lumen. The cavity caused the nucleus to move in the basal cytoplasm, which contained some mitochondria and rough endoplasmic reticulum cisternae. The basal cell membrane did not form any folds. Among endocrine cells, 2 types were distinguished: vesicular and granular. Regenerative cells were small cells with an oval nucleus, numerous mitochondria, and some rough endoplasmic reticulum cisternae (Fig. 1D).

### Changes in the Midgut Epithelium Ultrastructure in *S. exiqua* Larvae

The cytoplasm of goblet, endocrine, and regenerative cells showed no changes in any experimental groups of all generations (1–6, 12, and 18). Thus, the description below concerns only columnar cells. TEM allowed electron-dense glycogen granules to be distinguished from spheres with the remaining reserve material. Changes in the number of glycogen granules were observed in the cytoplasm of columnar cells of insects exposed to cadmium, while the presence and distribution of all organelles appeared to be similar to those in control organisms. In generations 1–6, no glycogen granule accumulation was detected in groups Cd11 and Cd22, while some small groups of glycogen granules were observed in the KCd and Cd44 groups. Glycogen granules were detected in all experimental groups in generations 12 and 18. We did not observe any changes in the distribution or accumulation of the reserve materials in the form of spheres, the only changes detected were in glycogen granules (Table 1).

**Table 1.** The accumulation of glycogen granules and spheres of the reserve material in the cytoplasm of columnar cells in experimental groups in generations I–VI, XII, and XVIII

Generation	Reserve material	KCd	5.5	11	22	44	K
I	Glycogen granules	+/−	+	−	−	+/−	+
	Spheres with reserve material	+	+	+	+	+	+
II	Glycogen granules	+/−	+	−	−	+/−	+
	Spheres with reserve material	+	+	+	+	+	+
III	Glycogen granules	+/−	+	−	−	+/−	+
	Spheres with reserve material	+	+	+	+	+	+
IV	Glycogen granules	+/−	+	−	−	+/−	+
	Spheres with reserve material	+	+	+	+	+	+
V	Glycogen granules	+/−	+	−	−	+/−	+
	Spheres with reserve material	+	+	+	+	+	+
VI	Glycogen granules	+/−	+	−	−	+/−	+
	Spheres with reserve material	+	+	+	+	+	+
XII	Glycogen granules	+	+	+	+	+	+
	Spheres with reserve material	+	+	+	+	+	+
XVIII	Glycogen granules	+	+	+	+	+	+
	Spheres with reserve material	+	+	+	+	+	+

### Autophagy in the Midgut Epithelium in *S. exiqua* Larvae: Microscopic Analysis

**Fig. 2.**
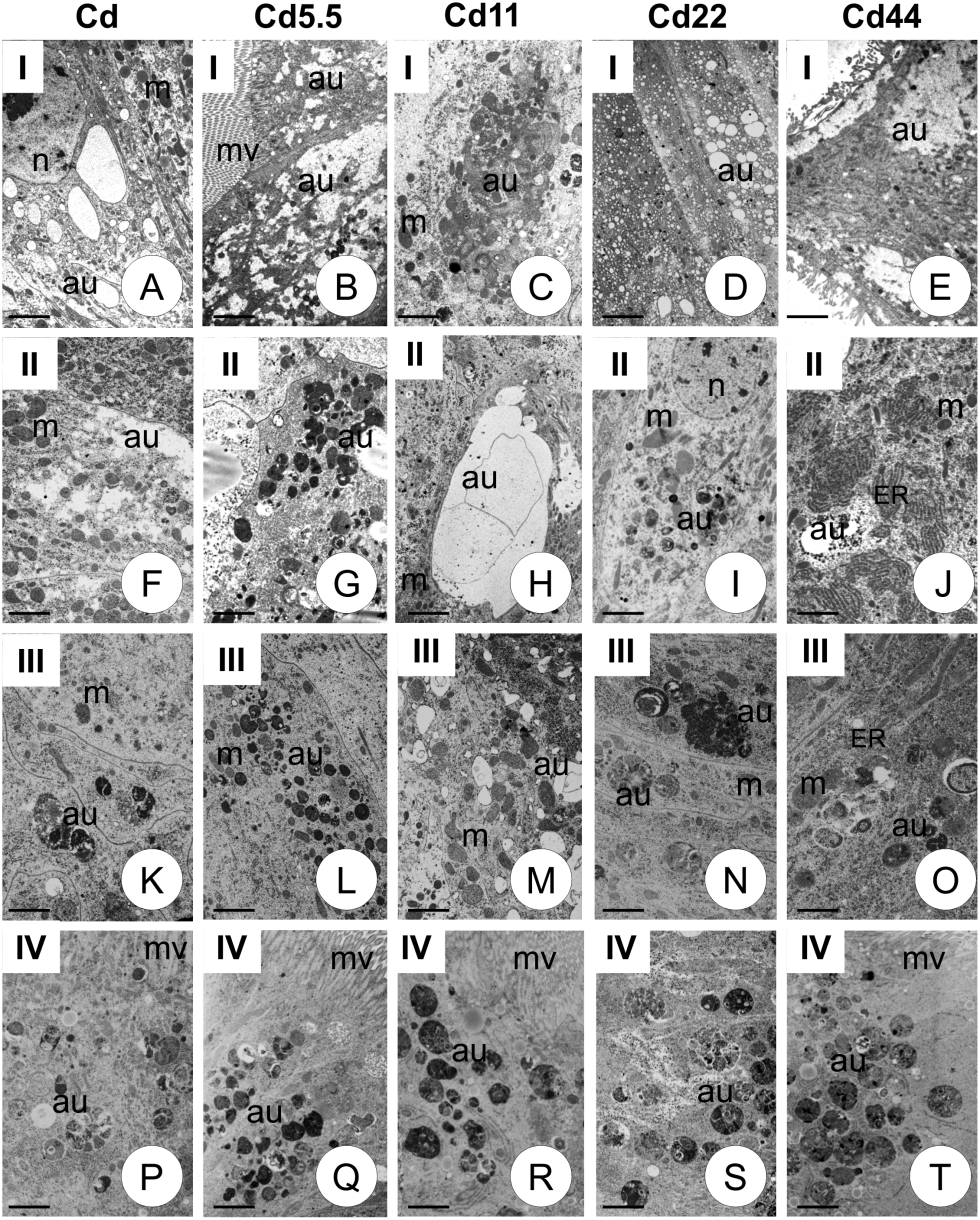
Autophagic structures: autophagosomes (au), autolysosomes (al), residual bodies (rb). in all experimental groups (Cd, Cd5.5, Cd11, Cd22, Cd44) and generations I–IV in the cytoplasm of columnar cells of *S. exiqua* V instar larvae. Mitochondria (m), endoplasmic reticulum (ER), microvilli (mv), nucleus (n). TEM. (A) Scale bar = 10 µm. (B) Scale bar = 10 µm. (C) Scale bar = 7 µm. (D) Scale bar = 10 µm. (E) Scale bar = 10 µm. (F) Scale bar = 8 µm. (G) Scale bar = 8 µm. (H) Scale bar = 10 µm. (I) Scale bar = 6 µm. (J) Scale bar = 7 µm. (K) Scale bar = 5 µm. (L) Scale bar = 10 µm. (M) Scale bar = 5 µm. (N) Scale bar = 5 µm. (O) Scale bar = 6 µm. (P) Scale bar = 6 µm. (Q) Scale bar = 8 µm. (R) Scale bar = 6 µm. (S) Scale bar = 6 µm. (T) Scale bar = 5 µm.

**Fig. 3.**
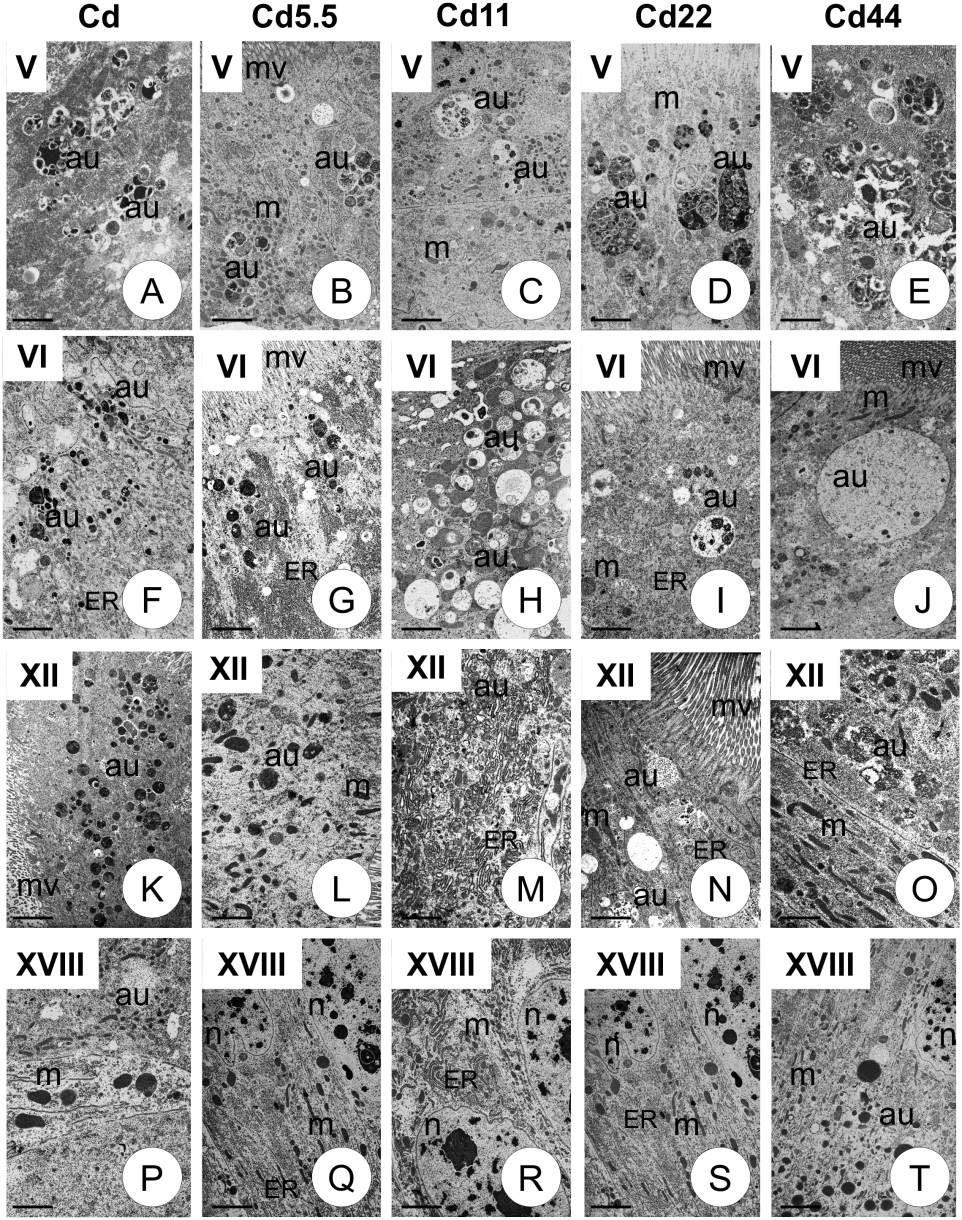
Autophagic structures (au) in all experimental groups (Cd, Cd5.5, Cd11, Cd22, Cd44) and generations V–VI, XII, XVIII in the cytoplasm of columnar cells of *S. exiqua* V instar larvae. Mitochondria (m), endoplasmic reticulum (ER), microvilli (mv), nucleus (n). TEM. (A) Scale bar = 6 µm. (B) Scale bar = 6 µm. (C) Scale bar = 8 µm. (D) Scale bar = 5 µm. (E) Scale bar = 5 µm. (F) Scale bar = 11 µm. (G) Scale bar = 8 µm. (H) Scale bar = 7 µm. (I) Scale bar = 8 µm. (J) Scale bar = 10 µm. (K) Scale bar = 10 µm. (L) Scale bar = 8 µm. (M) Scale bar = 6 µm. (N) Scale bar = 8 µm. (O) Scale bar = 6 µm. (P) Scale bar = 8 µm. (Q) Scale bar = 10 µm. (R) Scale bar = 8 µm. (S) Scale bar = 10 µm. (T) Scale bar = 8 µm.

TEM analysis enables all autophagic structures (autophagosomes, autolysosomes, and residual bodies) to be distinguished, as well as their localization in precise types of epithelial cells and their cytoplasmatic regions. The autophagic structures (autophagosomes, autolysosomes, residual bodies) were characterized by a double membrane and numerous cellular organelles inside. In addition, the form of autophagy (selective or nonselective) could be distinguished. Autophagy was observed only in the cytoplasm of columnar cells and occurred along the entire length of the midgut. Therefore, qualitative and quantitative analyses of autophagic structures were performed in columnar cells. TEM analysis revealed that autophagic structures mainly occurred in the apical cytoplasm, just beneath the cortical layer, but were also observed in the perinuclear region in all specimens examined (Figs. 2 and 3). No autophagic structures were detected in the cytoplasm of regenerative, goblet, or endocrine cells.

**Fig. 4.**
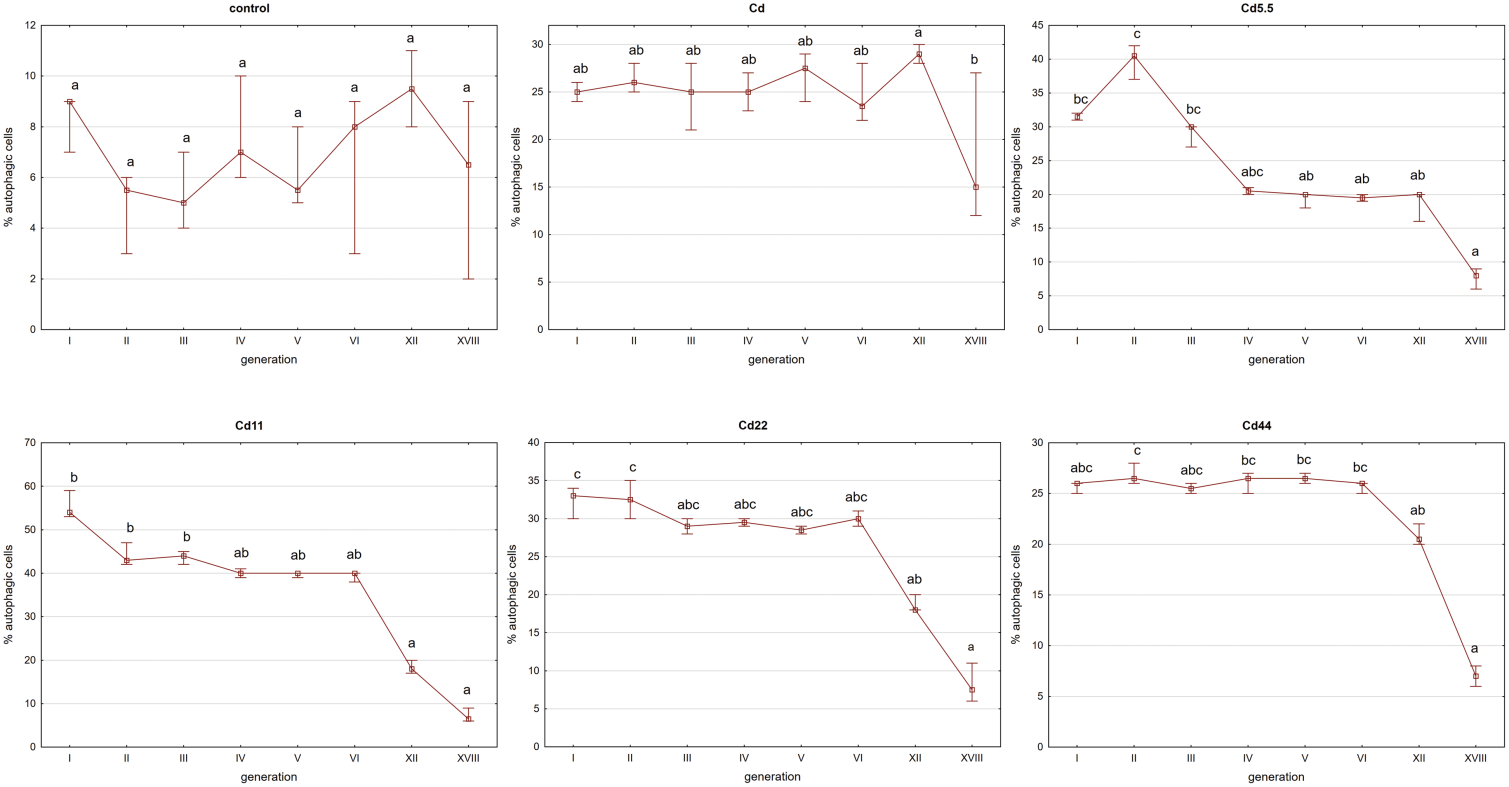
Total autophagic cells ratio (%) in midgut cells of V instar *S. exigua* larvae from I–VI, XII, and XVIII generations of insects from experimental and reference strains. Squares: median values, whiskers: 25–75%. Different letters (a, b, c) denote statistically significant differences between generations (ANOVA, Kruskal–Wallis H test, *P* < 0.05).

**Fig. 5.**
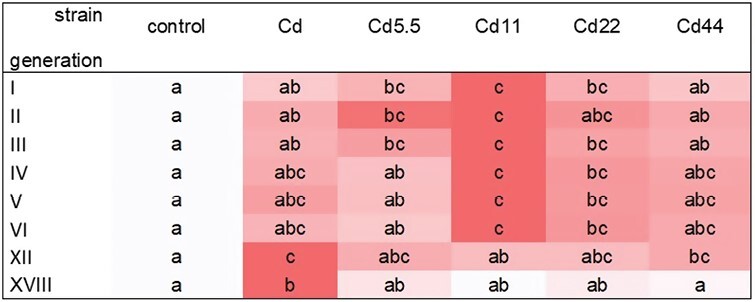
Total autophagic cells ratio in midgut cells of V instar *S. exigua* larvae from I–VI, XII, and XVIII generations of insects from experimental and reference strains. Values are expressed as the color scale, where dark shadow is the highest and white – the lowest. Different letters (a, b, c) denote statistically significant differences between strains (ANOVA, Kruskal–Wallis H test, *P* < 0.05).

Qualitative analysis of TEM observations revealed that, in strains exposed to cadmium for a maximum of 18 generations, autophagy levels at the beginning of the exposure (generations 1–2) were generally significantly higher than those observed after several additional generations (the accumulation of autophagic structures in the apical and perinuclear cytoplasm). Significant differences between the 2 first and subsequent generations began at generation 6 (strain, Cd5.5) or 12 (strains, Cd11, Cd22, and Cd44). In all experimental strains, autophagy levels in midgut cells from individuals of all exposure strains were generally significantly lower after 18 generations than in the first 2 generations (Fig. 4). TEM revealed that autophagic structures accumulated mainly in the apical cytoplasm of columnar cells. In between-strain comparisons, autophagy levels in midgut cells from individuals of the final studied generation of the experimental strains did not differ from controls, unlike those from earlier generations. Autophagy levels in midgut cells of individuals from generations 1–12 were significantly higher than those in controls of corresponding generations. The patterns of these differences varied in relation to the intensity of the pressure (Cd concentration in the diet). For experimental strains exposed to intermediate Cd concentrations (i.e., Cd11 and Cd22) after generation 12, levels were the same as those in the reference strains. Differences between reference and experimental strains exposed to lower cadmium concentrations (Cd5.5) appeared insignificant by the third generation, while those exposed to higher cadmium did not differ, even in cells from individuals from the 1st generation after exposure (Fig. 5). TEM analysis showed that various organelles and structures were enclosed inside the autophagosomes, i.e., cisterns of the endoplasmic reticulum, mitochondria, membrane fragments of various organelles, small vacuoles, etc. thus, only nonselective autophagy was present in all analyzed generations.

### Apoptosis and Necrosis in Midgut Epithelium of *S. exiqua* Larvae

**Fig. 6.**
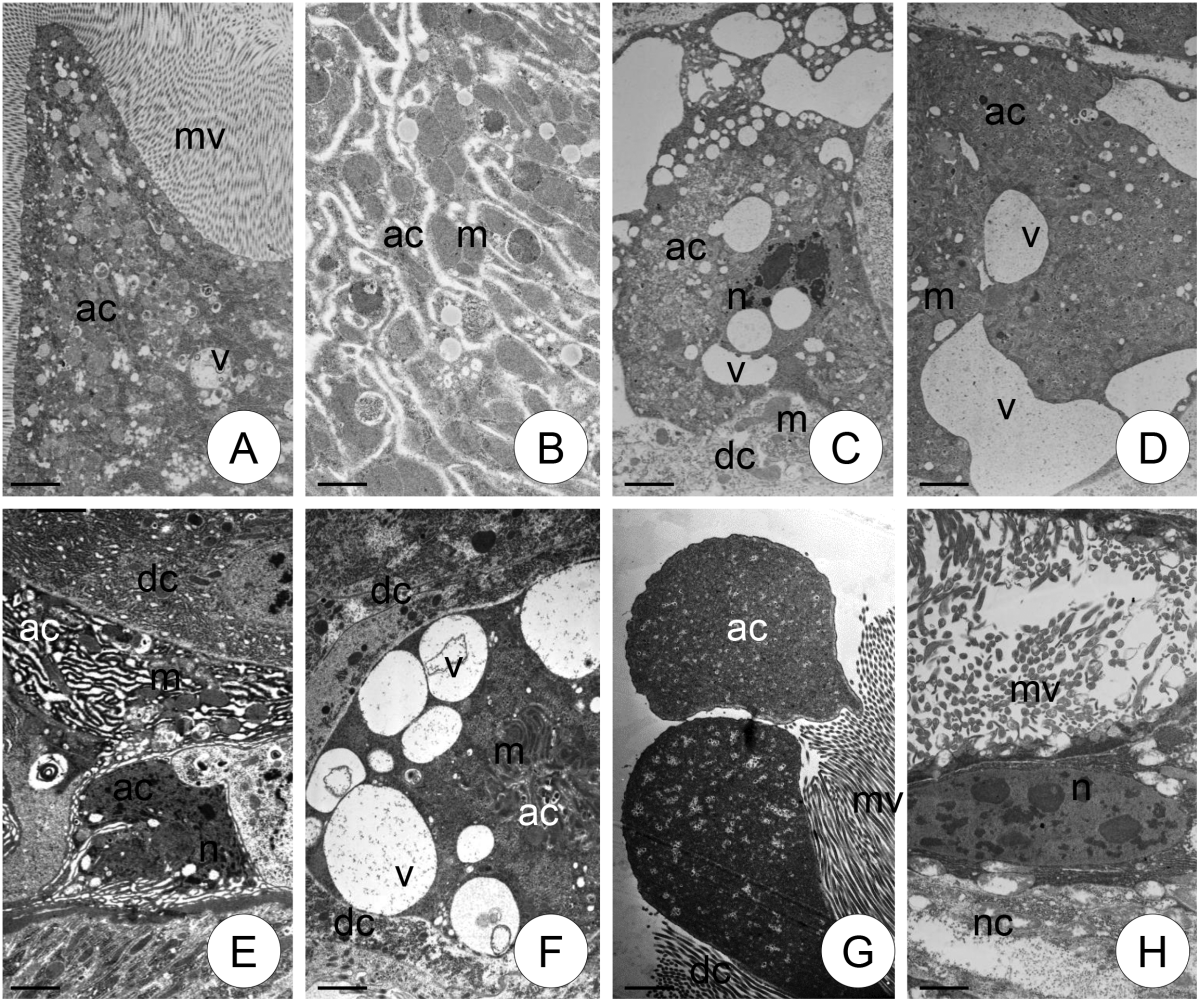
Apoptotic cells (ac) among columnar cells (dc) in the midgut epithelium of different experimental groups and different generations. Mitochondria (m), nucleus (n), microvilli (mv), vacuoles (v). TEM. (A) Cd22, V generation. (B–C) Cd22, III generation. (D) Cd22, VI generation. € Cd11, I generation. (F) Cd, V generation. (G) Cd44, I generation. (H) Necrotic cell with electron-lucent cytoplasm (nc). Cd, V generation. (A) Scale bar = 8 µm, (B) Scale bar = 5 µm, (C) Scale bar = 10 µm, (D) Scale bar = 10 µ€(E) Scale bar = 10 µm, (F) Scale bar = 8 µm, (G) Scale bar = 7 µm, (H) Scale bar = 8 µm.

Apoptosis was detected in columnar and goblet cells, while it was not detected in regenerative cells. At the beginning of apoptosis, the cell begins to shrink and extracellular spaces were observed among adjacent columnar cells. The cytoplasm became electron-dense, and mitochondria and endoplasmic reticulum cisternae were transformed (Fig. 6A–F). The nucleus became a lobular shape, and chromatin accumulated near the nuclear envelope, forming electron-dense patches characteristic of apoptosis (Fig. 6C and E). Shrunken apoptotic cells with the electron-dense cytoplasm lost contact with the basal lamina of the midgut epithelium, and were gradually released into the midgut lumen (Fig. 6G), where they were digested.

During necrosis, cells swelled and their cytoplasm became electron-lucent (Fig. 6H). The number of organelles decreased, and the apical cell membrane eventually ruptured, releasing all organelles into the midgut lumen.

**Fig. 7.**
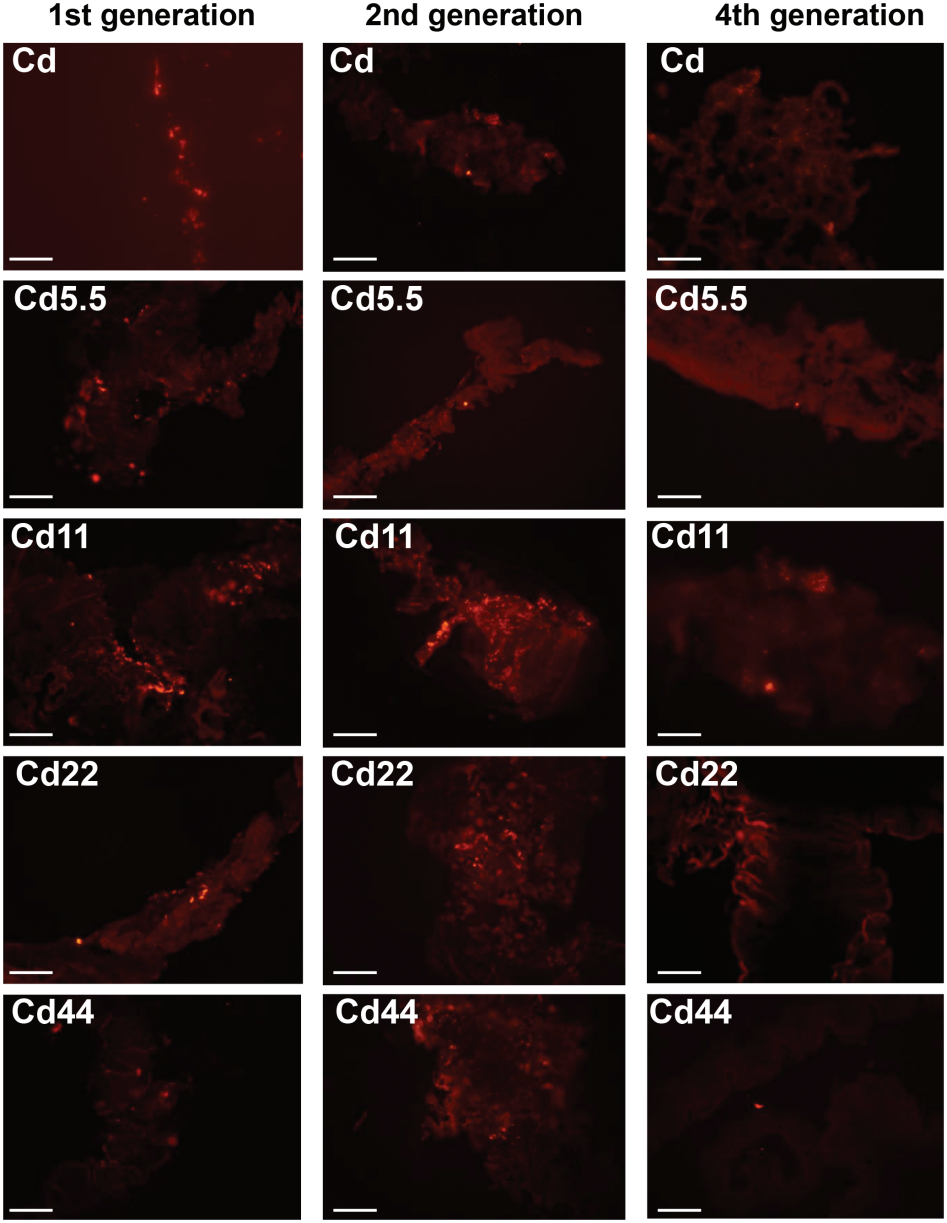
3D representation of the TUNEL assay of the midgut epithelium in *S. exiqua* larvae in all experimental groups (Cd, Cd5.5, Cd11, Cd22, Cd44). Nuclei of apoptotic cells (red signals). Cross sections. Confocal microscope. Scale bar = 150 µm.

DNA fragmentation, which occurs during apoptosis, was confirmed by TUNEL assay. Fluorescent microscopy qualitative analysis revealed that apoptotic cell signals in the midgut epithelium were strongest in individuals from the Cd11 strain. Signals in the Cd22 and Cd44 groups were slightly weaker than those in the Cd11 strain, but clearly stronger than those in the Cd5.5 and Cd strains. This relationship was observed in individuals from the first 3 generations, while from the 4th generation, the strength of apoptotic cell signals clearly decreased (Fig. 7). No signal was detected in midgut epithelium of specimens incubated without TdT enzyme solution as controls.

### Cytometric Analyses of Apoptosis

**Fig. 8.**
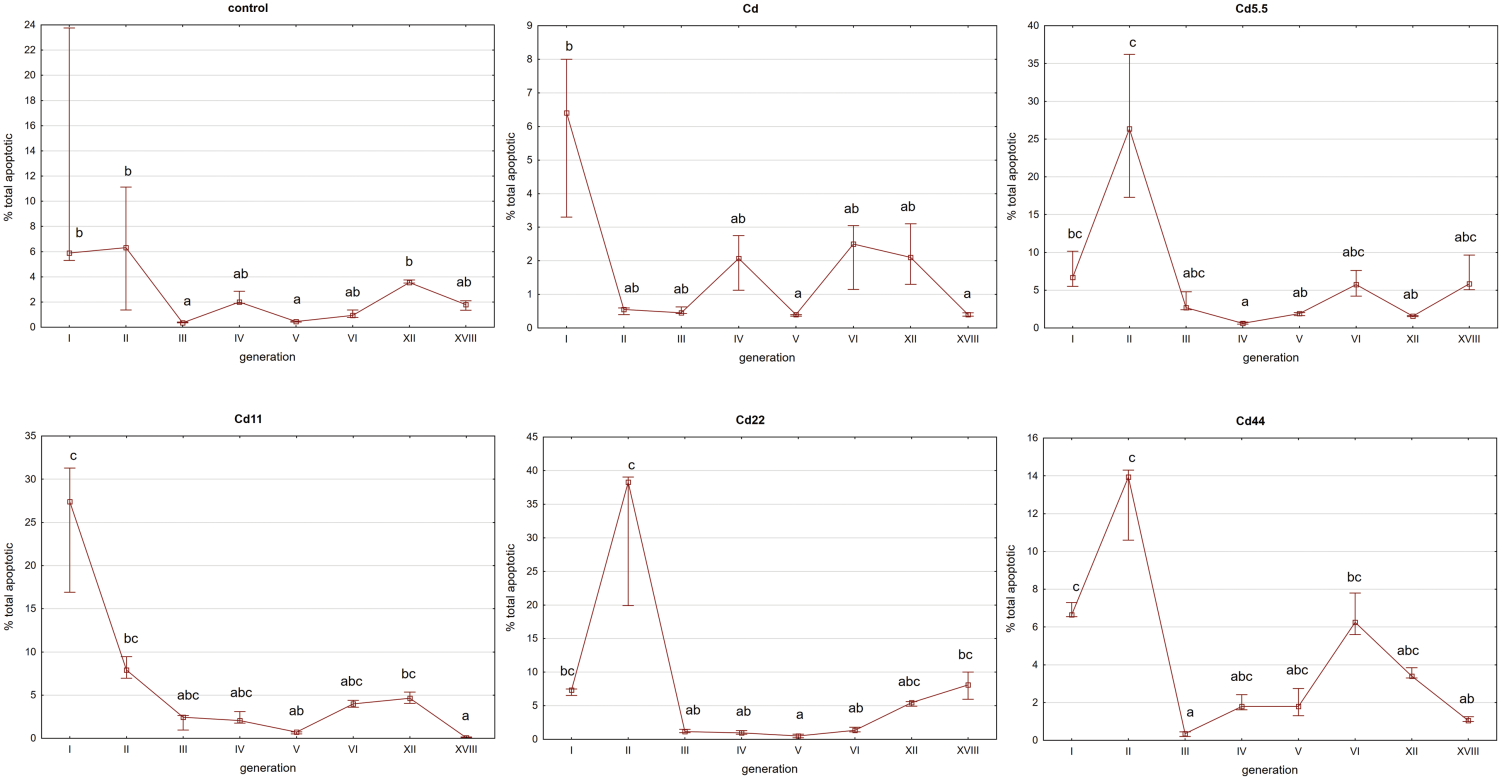
Total apoptotic cells ratio [%] in midgut cells of V instar *S. exigua* larvae from I–VI, XII, and XVIII generations of insects from experimental and reference strains. Squares: median values, whiskers: 25–75%. Different letters (a, b, c) denote statistically significant differences between generations (ANOVA, Kruskal–Wallis H test, *P* < 0.05).

In the reference control and Cd strains, no significant between-generation differences were generally detected, excluding the high median values (indicating high within-group variability) in the first generation. In the experimental strains, values were lower in generations 4 (Cd5.5), 5 (Cd11), and 3 (Cd22 and Cd44), relative to the first generation, After the threshold, values detected in the remaining generations did not generally differ significantly from one another (Fig. 8).

**Fig. 9.**
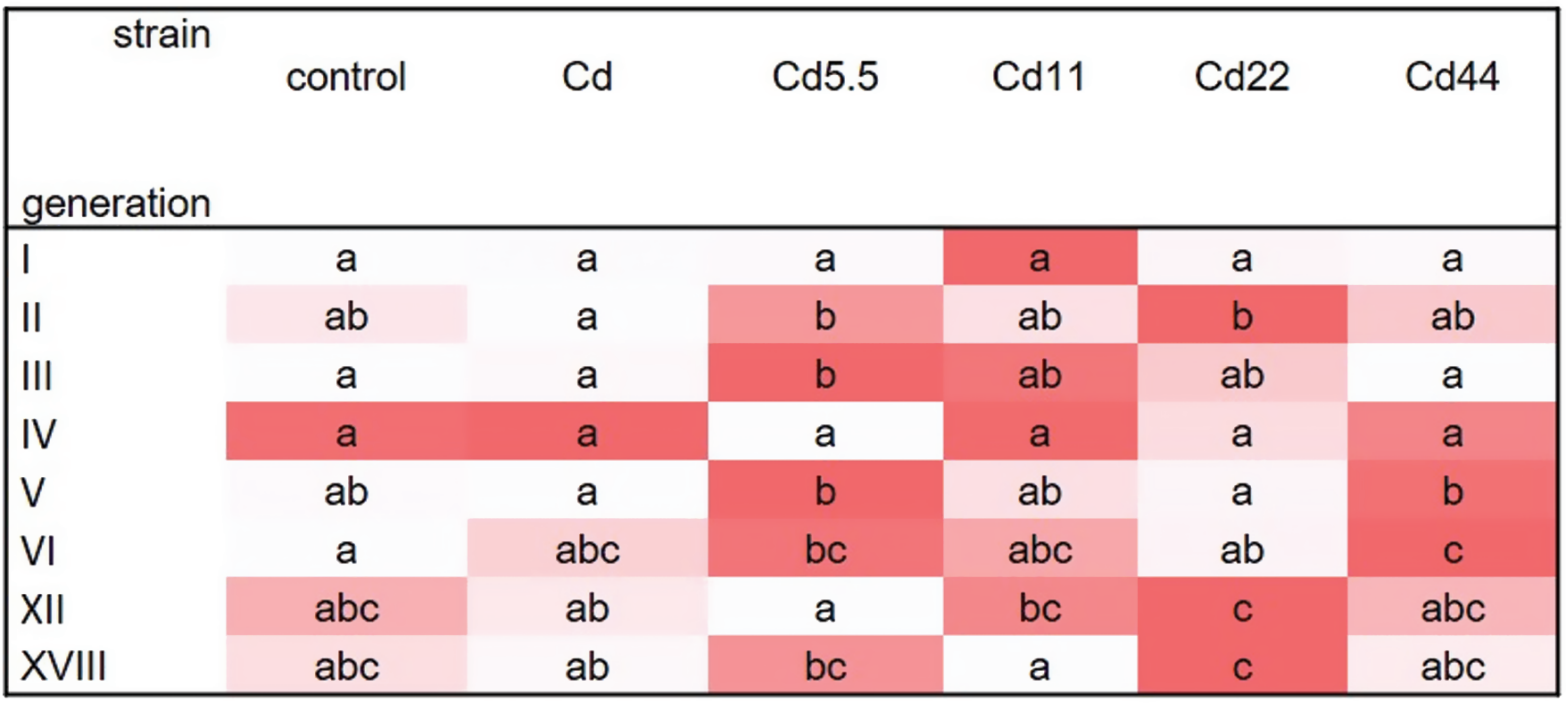
Total apoptotic cells ratio in midgut cells of V instar *S. exigua* larvae from I–VI, XII, and XVIII generations of insects from experimental and reference strains. Values are expressed as the color scale, where dark shadow is the highest and white is the lowest. Different letters (a, b, c) denote statistically significant differences between strains (ANOVA, Kruskal–Wallis H test, *P* < 0.05).

In between-strain comparisons, the only significant differences in midgut cell apoptosis levels in individuals from studied generations and reference strains were for the Cd5.5 strain in generations 3 and 6 and for the Cd44 strain in generation 3 (Fig. 9).

## Discussion

From an evolutionary perspective, anthropogenic stress, including chemical pollution, is a relatively new threat for living organisms. Therefore, defensive mechanisms at various biological levels can be regarded as side functions of reactions evolved to ensure the proper operation of physiological processes. Programmed cell death pathways are processes that control organism development and provide a counterbalance between normal and excessive cell proliferation (Gonçalves et al. 2018).

Based on the results obtained in this experiment, we conclude that, in response to an intense stressor, cell death processes are activated in a similar way to other mechanisms regarded as detoxifying. The initial contact with cadmium in food (first generation of insects in strains Cd5.5, Cd11, Cd22, and Cd44) resulted in significantly higher autophagy intensity than in the reference (control and Cd) strains. Levels of apoptosis intensity in this generation could not be accurately compared, due to high intragroup variability. In subsequent generations, elevated levels of cell death were either maintained or diminished, depending on the intensity of the pressure (Cd concentration in food). This was evident for apoptotic pathways, levels of which decreased in the 4th and 5th generations of insects exposed to lower Cd concentrations (strains Cd5.5 and Cd11), but in the 3rd generation of those exposed to higher Cd concentrations (strains Cd22 and Cd44). Finally, both autophagy and apoptosis intensity decreased, irrespective of the assessment method used, eventually reaching the levels detected in insects from the reference control and Cd strains. Thus, the results of our apoptosis intensity analysis agree with our previous findings, where metallothionein and heat shock protein (HSP70) levels were used as indicators of defense process activation (Tarnawska et al. 2019), and DNA damage level was used to assess the extent of adverse effects on the genetic material (Augustyniak et al. 2020). In both this study and our previous experiments, we concluded “the worse, the better”; i.e., more intense pressure causes a faster selection of individuals tolerant of environmental stress; however, elevated autophagy intensity in the experimental groups also remained in subsequent generations, finally lowering by generation 12 or 18, with no relationship to Cd concentration in food. This thought-provoking finding warrants further investigation, considering the continuum between the autophagy and apoptosis processes and, to some extent, their common regulation (Thorburn 2008). Moreover, autophagy can, but does not always, lead to apoptotic cell death (Lockshin and Zahra 2004). It is possible that the autophagic pathway has a supportive role in cells during increased activation of defensive processes. According to Maiuri et al. (2007), autophagy is an adaptive response to different types of stress, that protects cells against death. These authors also indicated that autophagy may promote adaptation to stress. “Stress” here referred to starvation, but, as mentioned in the introductory section and analyzed by Babczyńska et al. (2020), defense against stress is energy-consuming, requiring additional energy resources, as well as a trade-off between protection and other energy-demanding processes. Transmission electron microscopy can depict organelles or structures enclosed inside autophagic structures (Jung et al. 2019). Thus the type of autophagy can explain many mechanisms which could be activated against any stressor. Thus, mitophagy, reticulophagy, lipophagy, nucleophagy, peroxyphagy, etc. are treated as the selective form of autophagy (Schrader and Fahimi 2008, Chen et al. 2017, Rost-Roszkowska et al. 2019, Tarique et al. 2019). In this way, specific organelles are degraded, the excess of which, caused, for example, by a stressor, must be removed from the cell. As a consequence, the inflammatory process will not be activated. On the other hand, if various cellular organelles are neutralized within the autophagosome, nonselective autophagy is activated (Chen et al. 2017, Rost-Roszkowska et al. 2019). Our studies showed the presence of only nonselective autophagy, which indicates that the degradation of various damaged cellular structures allowed the cells to be protected from the activation of inflammation.

Tolerance may be based on either heritable genetic changes or phenotypic plasticity. Together with the results of this study, the findings presented by Tarnawska et al. (2019), Augustyniak et al. (2020), and Babczyńska et al. (2020) reflect the results of a larger project examining the dilemma between microevolution (inheritable changes) and broader tolerance (plasticity). As concluded in previous reporting analyses of heat shock proteins, DNA stability, and autophagy-based defense, observed tolerance to cadmium, at least within the studied period of 36 generations, is due to phenotype plasticity; however, only overview from a broader perspective can support this conclusion. Although separate analyses of individual markers point to phenotypic plasticity, combined assessment of these findings strongly support this inference. According to a review by Moczek (2010), phenotypic plasticity enables organisms to adjust to a range of environmental conditions, and is an individual trait that determines the range of an individual’s gene expression in response to stress. Therefore, within the population, there will be individuals with different dominant defense mechanisms. This may explain the wide intragroup variability detected, especially during the initial exposure stage. Phenotype frequencies may change from generation to generation in response to environmental conditions and affect the intra-generation phenotype expression of individuals (Rodrigues and Beldade 2020). The authors explain the mechanisms underlying thermotolerance in insects, taking into account the expression of temperature-regulated genes, including genes responsible for temperature-dependent phenotypes, such as body pigmentation, body size, and HSP levels, among others. Analogous analysis has also been conducted under other types of pressure; for example, Bicho et al. (2017) conducted a multigenerational study on nanoparticle toxicity to the earthworm, *Enchytraeus crypticus*, and found that worms exhibited increased tolerance to CuCl_2_, attributable to plasticity-derived physiological adaptation.

Based on the results of this and previous studies, phenotypic plasticity appears to manifest itself in the simultaneous occurrence of various processes, where the participation of each mechanism is an individual feature of an efficient defense against chemical stress. The overall picture and intragroup variability of a population result from the overlapping characteristics of each individual. Variations in individual response may appear beneficial to the population, allowing selection of the most tolerant individuals under constant environmental stress. Different mechanisms are induced simultaneously, and act in complementary ways, according to different patterns. In this project, various reactions were activated and expired over different periods of time, although the modes of action (autophagy and apoptosis) appeared to be similar. The pattern of changes in apoptosis intensity was comparable to that in DNA damage reported, with reference levels reached by the fourth generation (Augustyniak et al. 2020), while autophagy, similar to stress protein levels (Tarnawska et al. 2019), achieved levels comparable to reference strains after 12 generations or more. This correlation between autophagy intensity and stress protein levels may be connected with the fact that both processes can be activated by oxidative stress (Viarengo et al. 2000, Dukić-Ćosić et al. 2020). The pro-oxidative properties of cadmium are maintained throughout the exposure period, necessitating constant antioxidative defense. It is possible that an antioxidative pathway, efficient enough to prevent stress protein synthesis and autophagy induction, develops after 12 generations. Alternatively, autophagy may be necessary to assure energy supplies for stress protein synthesis for as long as it is induced, which is supported by the results described in Babczyńska et al. (2020), demonstrating that autophagy is activated under energy-demanding conditions.

Our analysis clearly demonstrates that apoptosis and autophagy do not proceed in parallel from generation to generation. High autophagy levels were maintained for more generations (minimum, 12 generations) beginning with the first generation exposed to cadmium; autophagy levels only decreased to the control level (below 10%) in the 18th generation. Apoptosis reached the basal levels measured in the control strain much earlier; at the third generation for insects under the highest Cd pressure (Cd44), or the fourth (Cd5.5) and fifth (Cd11 and Cd22) generations in strains with lower exposure. The cross-talk between these 2 processes suggests a potentially protective role of autophagy in preventing apoptotic cell death. This kind of interplay has been described by other researchers, mainly in the context of human or other mammalian cell lines, and often in cancer cells. In general, relationships between autophagy and apoptosis are described as complex (Maiuri et al. 2007, Nikoletopoulou et al. 2013, Mariño et al. 2014). Maiuri et al. (2007) and Mariño et al. (2014) reported that autophagy usually blocks apoptosis induction, thereby increasing stress tolerance under certain circumstances and enabling the organism to avoid stress-related death. This type of interplay most likely explains the interdependence between autophagy and apoptosis observed in this study. Studies on cell destructive processes in response to environmental stressors are, unfortunately, rare, particularly in relation to metals. A recent report on the effects of exposure of honeybees to imidacloprid revealed parallel induction of both pathways ([Bibr CIT0008][Bibr CIT0008]). Further, Maiuri et al. (2007) indicated that, under specific conditions, autophagy can stimulate apoptosis. In this example, these specific conditions may refer to the level of pressure; that is, the dose or concentration the animal is exposed to (e.g., lethal or sublethal doses of a toxin).

In discussing these results, it is necessary to acknowledge the ideal (to a large extent) experimental set-up, particularly if selection in response to a factor is to be investigated. Benefits include simplification, relative to natural field conditions, and the exclusion of simultaneous additional interacting factors; this assumption was the basis of the multiannual project of which this report is a part; however, this may represent a limitation for further study, due to gene pool depletion, and narrowing of phenotypes, potentially leading to lethal effects of inbreeding.

In conclusion, elevated levels of programmed cell death intensity in response to cadmium decreased after several generations in *S. exiqua*, indicating tolerance of individuals to cadmium in the diet, which partly supported both hypotheses tested in this study. However, the number of generations depended not only on the intensity of pressure, but also on the pathway that cells entered in response to prolonged chemical pressure.

## Data Availability

The datasets generated and/or analyzed during the current study are available from the corresponding author on reasonable request.
